# Adherence challenges with daily oral pre‐exposure prophylaxis during pregnancy and the postpartum period in South African women: a cohort study

**DOI:** 10.1002/jia2.26044

**Published:** 2022-12-08

**Authors:** Dvora Joseph Davey, Dorothy C. Nyemba, Jose Castillo‐Mancilla, Lubbe Wiesner, Jennifer Norman, Rufaro Mvududu, Nyiko Mashele, Leigh F. Johnson, Linda‐Gail Bekker, Pamina Gorbach, Thomas J. Coates, Landon Myer

**Affiliations:** ^1^ Division of Infectious Diseases Geffen School of Medicine University of California Los Angeles Los Angeles California USA; ^2^ Division of Epidemiology and Biostatistics School of Public Health University of Cape Town Cape Town South Africa; ^3^ The Desmond Tutu Health Foundation University of Cape Town Cape Town South Africa; ^4^ Division of Infectious Diseases School of Medicine University of Colorado‐Anschutz Medical Campus Aurora Colorado USA; ^5^ Division of Clinical Pharmacology Department of Medicine University of Cape Town Cape Town South Africa; ^6^ Centre for Infectious Disease Epidemiology and Research University of Cape Town Cape Town South Africa

**Keywords:** adherence, breastfeeding, pre‐exposure prophylaxis, pregnant, PrEP, South Africa

## Abstract

**Introduction:**

Daily oral pre‐exposure prophylaxis (PrEP) can reduce HIV acquisition. However, prevention effectiveness requires daily adherence prior to and during periods of sexual activity. Little is known about pharmacologic measures of PrEP adherence during pregnancy and postpartum and the factors related to optimal adherence during periods of sexual activity in this population.

**Methods:**

Between August 2019 and October 2021, we enrolled pregnant women without HIV at their first antenatal care visit followed‐up through 12 months postpartum. Eligible women ≥16 years old received HIV prevention counselling and were offered oral PrEP (TDF‐FTC). We quantified tenofovir‐diphosphate (TFV‐DP) in dried blood spots in women who reported taking PrEP in the past 30 days (at quarterly follow‐up visits). We used regression models with generalized estimating equations to evaluate correlates of TFV‐DP (any vs. none, and ≥2 vs. <2 doses/week), adjusting for maternal age and pregnancy status.

**Results and discussion:**

In 382 women who started PrEP in pregnancy, returned for follow‐up and reported PrEP use in the past 30 days, the median age was 27 years (interquartile range [IQR] = 23–32), and the median time on PrEP was 168 days (IQR = 84–252 days). Half of the samples had quantifiable TFV‐DP at any time point (52%), declining from 67% of pregnant women 3 months post‐initiation to 31% of postpartum women by 12 months. Overall, 72% had concentrations corresponding to <2 doses/week; 25% ≥2 doses/week; 3% 7 doses/week. Concentrations were lower in postpartum versus pregnancy (age‐adjusted odds ratio [aOR] = 0.44; 95% confidence interval [CI] = 0.35–0.54). The correlation of self‐reported adherence and TFV‐DP ranged from –0.07 in pregnancy to 0.25 in postpartum women. Variables associated with having quantifiable TFV‐DP included partner living with HIV/unknown serostatus (aOR = 1.50; 95% CI = 1.01–2.22), and reported frequency of sexual activity in the past month (aOR sex >5/month vs. no sex or <5 times/month = 2.11; 95% CI = 1.58–2.82) adjusting for age and pregnancy versus postpartum status. TFV‐DP concentrations declined over follow‐up time (aOR for 6 vs. 3 months = 0.49; 95% CI = 0.36–0.67).

**Conclusions:**

Objectively measured adherence to PrEP was low overall and did not correlate with self‐reported use. There is an urgent need for objective adherence measures to support clinical decision‐making as well as adherence support interventions as part of PrEP services for pregnant and postpartum women at risk of HIV.

## 1 INTRODUCTION

Oral pre‐exposure prophylaxis (PrEP) with daily tenofovir disoproxil fumarate (TDF) and emtricitabine (FTC) is effective at reducing sexual HIV acquisition [1, 2, 3]. However, prevention effectiveness is reliant on daily adherence prior to and during periods of sexual activity [4, 5, 6, 7]. Little is known about pharmacologic adherence measures during pregnancy and postpartum and about the factors related to optimal adherence during periods of sexual activity [8]. Prior studies demonstrated that pregnant and breastfeeding women (PBFW) using PrEP may struggle with daily use and continuation because of various personal, interpersonal or healthcare access barriers [9, 10, 11].

Adherence to daily oral TDF‐based PrEP may be especially important for pregnant women because of lower concentrations of tenofovir diphosphate (TFV‐DP) during gestation [12], highlighting the importance of understanding daily adherence and factors that affect daily adherence in PBFW. The dose–response relationship of daily PrEP use on the prevention of HIV infection is most evident when adherence is measured by assessing intracellular levels of TFV‐DP, the active anabolite of tenofovir, in red blood cells, which are abundant in dried blood spots (DBS) [13]. Further, while self‐reported adherence is commonly assessed in routine clinical care, studies in women have provided evidence of discordance between self‐reported adherence and objective measures of adherence [14]. There is limited research on how best to measure PrEP adherence in PBFW and factors related to prevention‐effective adherence. To address these gaps, we aimed to quantify PrEP use by measuring TFV‐DP in DBS in a cohort of PBFW who reported recent oral PrEP use.

## 2 METHODS

The PrEP in Pregnant and Postpartum women (PrEP‐PP) study was a prospective cohort in which we enrolled consenting pregnant, adolescent girls and women (age ≥16 years) without HIV at the first antenatal care (ANC) visit. We followed participants through 12 months post‐delivery from one public health clinic in Cape Town, Western Cape, South Africa.

### 2.1 Study participants

PrEP‐PP recruitment began in August 2019 and concluded in October 2021. Study methods are described previously [15]. For this analysis, we included women who reported taking PrEP in the last 30 days before the study visit, during quarterly visits over pregnancy and postpartum (through 12 months postpartum). Eligible consenting participants received the equivalent of approximately $8 USD in grocery vouchers for their time and effort in the study as well as remuneration for transportation costs independent of their PrEP use.

### 2.2 Data collection

At each visit, participants received individual counselling about HIV prevention while pregnant and breastfeeding, including PrEP. Study interviewers conducted a survey at each study visit, which took 15–20 minutes using REDCap, a secure web‐based platform [16]. If the participant decided to start PrEP, the study nurse provided the participant with a 1‐month supply of TDF‐FTC and an invitation card to return in 1 month for follow‐up testing (after which participants received a 3‐month prescription to correspond with quarterly study follow‐up visits). At each visit, the study interviewer asked participants about missed doses in the last 7 and 30 days, with additional questions on the reasons for missing doses. We collected blood for DBS in women who reported taking PrEP in the last 30 days prior to the visit, as our cohort included women who cycled on and off PrEP.

TFV‐DP in DBS, a measure of cumulative TDF adherence [17, 18, 19], was quantified using a validated liquid chromatography‐tandem mass spectrometry assay at the Division of Clinical Pharmacology, University of Cape Town [12, 20]. The lower limit of quantification for TFV‐DP was 16.6 fmol/3‐mm punch. The thresholds used to establish adherence were derived from the “Pharmacokinetic of Oral PrEP during Pregnancy and Postpartum” Trial, stratified by pregnancy versus postpartum [21].

### 2.3 Statistical analyses

We analysed the proportion of women who continued PrEP after 3, 6, 9 and 12 months based on self‐report and the corresponding concentrations of TFV‐DP in DBS in women who returned for the study visit between 3 and 12 months after starting PrEP and reported using PrEP in the past month. We evaluated the correlation between self‐reported adherence and the concentrations of TFV‐DP stratified by pregnancy versus postpartum for 3‐ and 6‐month visits and postpartum only for 9‐ and +12‐month visits. We constructed logistic regression models to estimate associations between selected risk factors and PrEP adherence measures; throughout the models used the method of generalized estimating equations to account for the intra‐individual clustering of measures. We developed separate models to predict: (1) any TFV‐DP present (yes/no), and (2) levels of TFV‐DP equivalent to ≥2 estimated doses/week compared with <2 doses/week by pregnancy versus postpartum, in the subset of measurements with detectable TFV‐DP. We adjusted for maternal age at baseline and pregnancy versus postpartum status in individual models. In models of TFV‐DP and sexual activity, we adjusted for maternal age, gestational age at baseline, relationship status and pregnancy versus postpartum status. All statistical analyses were conducted with STATA v.14 [22].

### 2.4 Ethics

Participants provided written consent and the study was approved by the Human Research Ethics Committee of the University of Cape Town Faculty of Health Sciences (#297/2018) and by the University of California, Los Angeles Institutional Review Board (IRB#18‐001622).

## 3 RESULTS AND DISCUSSION

**Figure 1:**
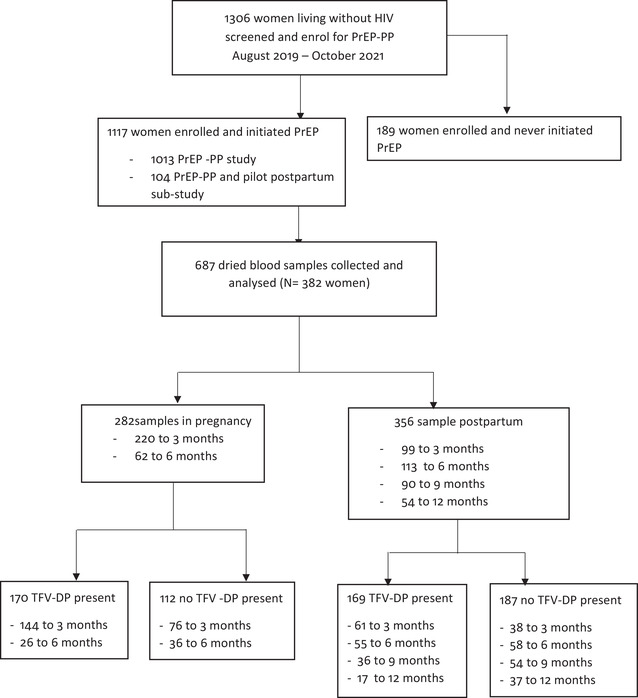
Consort flow diagram for pregnant women enrolment in PrEP‐PP study and eligible for dried blood spot analyses of tenofovir diphosphate (TFV‐DP).

We collected and analysed the DBS in women who returned for the study visit between 3 and 12 months after starting PrEP and reported PrEP use in the past month (*n* = 382 of 1200 enrolled in the cohort; 32%; Figure 1). The median age was 27 years (interquartile range [IQR] = 23–32), and over half were >20 weeks gestation at the first antenatal visit (*n* = 206; 54%). The median time on PrEP was 168 days (IQR = 84–252 days). Overall, we analysed samples from 54% of women in the cohort at the date of analysis (687 of 1282 samples available as of the study date). Women who missed a study visit or did not report taking PrEP in the last 30 days did not have a sample drawn.

In 687 DBS samples from 382 women, 360 (52%) had detectable TFV‐DP levels. At the 3‐month visit, 57% of women were still pregnant (*n* = 183 of 319), and 81% reported taking PrEP every day, and 67% had any TFV‐DP detected. Overall, 37% of pregnant women had TFV‐DP concentrations that were ≥2 doses/week, and 7% met the TFV‐DP cut‐off that was consistent with taking ∼7 doses in the last week prior to the study visit (*n* = 14). Among postpartum women taking PrEP until 3 months, 71% reported taking PrEP daily and 60% had any TFV‐DP detected. Overall, 31% of samples were consistent with a recent PrEP intake of ≥2–6 doses/week, and 2% (*n* = 3) met the TFV‐DP cut‐off that was consistent with taking ∼7 doses in the week prior to the study visit (Table 1).

**Table 1:** Association between maternal socio‐demographic and health characteristics and likelihood of TFV‐DP in blood according to DBS analysis in pregnant and postpartum women (*n* = 382)

	*n* (%)	Unadjusted OR (95% CI)	*p*‐value	Adjusted OR^a^ (95% CI)	*p*‐value
**Socio‐demographic characteristics**					
Maternal age (median, IQR) years	**27 (23–32)**	**1.03 (0.99–1.06)**	**0.05**	**1.01 (1.00–1.02)**	**0.04**
16–24 years	135 (35)	ref		–ref	
25–29 years	116 (30)	0.79 (0.50–1.25)	0.32	0.69 (0.39–1.20)	0.18
≥ 30 years	131 (34)	**1.60 (1.02–2.50)**	**0.04**	–1.32 (0.67 0 2.60)	0.40
GA at booking (median, IQR) weeks					
< 20 weeks @ booking	176 (46)	ref		ref	
≥ 20 weeks @ booking	206 (54)	1.34 (0.92–1.94)	0.11	**1.59 (1.05–2.41)**	**0.02**
BMI at booking (median, IQR) kg/m^2^	31 (26–36)	1.01 (0.99–1.03)	0.26	1.01 (0.98–1.03)	0.35
Creatinine at booking (median, IQR) μmol/L	46 (41–52)	1.00 (0.98–1.02)	0.58		
Gravidity (median, IQR)	2 (1–3)	**1.14 (0.97–1.34)**	**0.09**	1.07 (0.84–1.35)	0.55
Pregnancy status^b^					
Antenatal	—‐	ref		ref	
Postpartum	—‐	**0.48 (0.36–0.63)**	**<0.01**	**0.43 (0.31–0.58)**	**<0.01**
**Education level completed**					
Primary	199 (52)	ref			
Secondary and tertiary	183 (48)	0.87 (0.60–1.26)	0.48		
**Relationship with father of child**					
Married/cohabiting	145 (38)	ref		ref	
Not married/not cohabiting	237 (62)	1.02 (0.70–1.50)	0.88	1.13 (0.72–1.79)	0.57
**Employment status**					
Full‐time employment	98 (26)	ref		ref	
Part‐time employment	29 (8)	1.51 (0.69–3.32)	0.29	1.84 (0.80–4.23)	0.14
Informal employment	2 (1)	0.56 (0.04–7.41)	0.66	0.49 (0.03–6.81)	0.59
Attending school/college	19 (5)	0.84 (0.35–2.02)	0.71	1.17 (0.44–3.14)	0.74
Unemployed/not studying	234 (61)	0.92 (0.60–1.41)	0.72	0.92 (0.57–1.49)	0.75
**Socio‐economic status (SES)**					
Low SES	124 (32)	ref			
Moderate/high SES	258 (68)	0.81 (0.54–1.21)	0.30		
**Psychosocial characteristics**					
Depression symptoms (EPDS)					
Below threshold <11	352 (92)	ref			
Above threshold > = 11	30 (8)	0.82 (0.42–1.59)	0.56		
Alcohol use prior current pregnancy					
Yes	24(6)	0.82 (0.42–1.59)	0.56		
No	358 (94)	ref			
Ever experienced IPV					
Yes	65 (17)	1.23 (0.75–2.01)	0.39		
No	317 (83)	ref			
Diagnosed with STI at enrolment					
Positive	130 (34)	1.16 (0.78–1.71)	0.44		
Negative	252 (66)	ref			
**Partner's serostatus** ^c^					
Concordant HIV negative	268 (70)	ref		ref	
Serodiscordant or unknown	114 (30)	**1.36 (0.97–1.92)**	**0.08**	**1.50 (1.01–2.22)**	**0.04**
**HIV risk perception at enrolment**					
No chance at all	208 (55)	ref		ref	
Some/high chance	174 (45)	1.32 (0.91–1.91)	0.13	1.27 (0.85–1.90)	0.23
**Sexual behaviour** ^c^					
**Any vaginal sex at baseline**					
No	17 (4)	ref			
Yes	365 (96)	0.70 (0.28–1.74)	0.45		
**Sexual activity in the past month** ^b^, ^c^					
Sex < 5 times and no sex	—	ref		ref	
Sex 5+ times	—	**2.89 (2.22–3.76)**	**<0.01**	**2**.**11 (1**.**58–2.82)**	**<0.01**
**Number of sex partners in the past 3 months**					
1 sex partner	371 (97)	ref			
2+ sex partners	11 (3)	0.78 (0.33–1.85)	0.57		
**Condom use during sex**					
Sometimes/always	251 (69)	ref			
Never	131 (31)	1.35 (0.90–2.02)	0.14		
**Prior knowledge of PrEP**					
No	279 (73)	ref			
Yes	103 (27)	1.02 (0.67–1.54)	0.90		
**Baseline attitude about PrEP**					
Fear about PrEP					
Yes	59 (15)	0.89 (0.53–1.49)	0.66		
No	323 (85)	ref			
Concerns about taking PrEP					
Yes	53 (14)	0.95 (0.55–1.62)	0.86		
No	329 (86)	ref			
Worried about side effects					
Very/somewhat worried	99 (26)	ref		ref	
Not worried	283 (74)	**0.60 (0.39–0.93)**	**0.02**	0.72 (0.45–1.16)	0.18
Interested in taking PrEP					
Not interested	38 (10)	ref			
Interested	344 (90)	1.21 (0.76–1.93)	0.41		
Perceived effectiveness of PrEP					
Not effective	66 (17)	ref			
Effective	316 (83)	1.03 (0.67–1.58)	0.88		
**Study visit**					
3 months	319 (84)	ref		ref	
6 months	175 (46)	**0.46 (0.35–0.61)**	**<0.01**	**0.49 (0.36–0.67)**	**<0.01**
9 months	90 (23)	**0.39 (0.26–0.56)**	**<0.01**	**0.46 (0.30–0.71)**	**<0.01**
12 months	54 (14)	**0.32 (0.20–0.52)**	**<0.01**	**0.39 (0.23–0.67)**	**<0.01**

Note: Bold *p*<0.10.

Abbreviations: ANC, antenatal care; EPDS, Edinburgh Postnatal Depression Scale; GA, gestational age; IQR, interquartile range; *n*, number of participants; OR, odds ratio; PrEP, pre‐exposure prophylaxis; SD, standard deviation.

^a^ Each individual model adjusted for maternal age at enrolment, pregnancy versus postpartum status.

^b^ Not static measures.

^c^ Adjusted for maternal age, gestational age, relationship status and pregnancy versus postpartum status.

**Figure 2:**
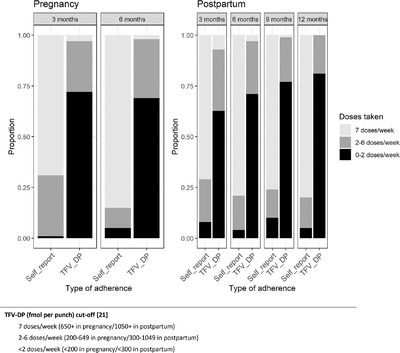
Estimated number of doses taken in the prior 7 days by study visit, pregnancy status, self‐report and TFV‐DP level in dried blood spots.

At the 6‐month visit (*n* = 175), 33% of women were still pregnant. Pregnant and postpartum women reported high levels of daily PrEP adherence (79% pregnant women and 76% postpartum women reported taking PrEP daily in the last week). Overall, 29% of pregnant women had TFV‐DP concentrations consistent with ≥2 doses/week, compared with 22% in postpartum women. At 9 months, all women were postpartum (*n* = 90), and three‐fourths reported taking PrEP daily in the past week (76%), and 23% of samples had TFV‐DP concentrations consistent with taking PrEP ≥2 days/week. Finally, through the 12‐months’ follow‐up, 80% of postpartum women reported taking PrEP every day in the past week, and 19% had TFV‐DP concentrations consistent with a PrEP intake of ≥2 doses/week. The correlation coefficients across study visits between self‐report responses and TFV‐DP in DBS ranged from –0.09 (95% CI = –0.35, 0.17) for postpartum women at the 12‐month study visit to 0.25 (95% CI = 0.05, 0.44) for postpartum women at the 9‐month study visit. Among the participants with both self‐reported PrEP adherence and DBS sampling, the percentage of women who reported taking 7 doses/week was consistently very high across all the visits (Figure 2).

### 3.1 Correlates of TFV‐DP in pregnant and postpartum women

Since the number of women with high TFV‐DP (consistent with 7 doses/week) was small (*n* = 14; 7% of samples), we focused on quantifiable TFV‐DP in our analysis. Specific participant, visit and behavioural characteristics were included in the multivariable model estimating the adjusted odds ratio (aOR) of having quantifiable TFV‐DP in DBS samples that were tested. Correlates of having quantifiable TFV‐DP present were older maternal age (aOR per year of age = 1.01; 95% CI = 1.00, 1.02) and being ≥20 weeks gestational age at first ANC visit (aOR = 1.59; 95% CI = 1.05, 2.41). In addition, being postpartum versus pregnant (aOR = 0.43; 95% CI = 0.31–0.58) was associated with lower recent PrEP adherence. Similarly, TFV‐DP was highest after 3‐months’ follow‐up and declined significantly during every quarterly visit (aOR for 6 vs. 3 months = 0.49; 95% CI = 0.36, 0.67, and aOR for 12 vs. 3 months = 0.39; 95% CI = 0.23, 0.67). There were similar declines in quantifiable TFV‐DP in postpartum women at 6 versus 12 months (aOR = 0.34; 95% CI = 0.22, 0.51).

HIV risk factors that were associated with having quantifiable TFV‐DP included a partner living with HIV or an unknown serostatus (aOR = 1.50; 95% CI = 1.01–2.22), and reported frequency of sexual activity in the month preceding the DBS collection. Women who reported having sex ≥5 times in the past month had over two times the odds of having any quantifiable TFV‐DP in DBS, compared to women who reported sex <5 times or no sex (aOR = 2.11; 95% CI = 1.58, 2.82) (Table 1). Breastfeeding postpartum women (vs. non‐breastfeeding postpartum women) had increased odds of having levels commensurate with ≥2 doses/week (aOR = 1.83; 95% CI = 1.04, 3.20).

Here, we found that daily PrEP use was suboptimal throughout the peripartum period and declined over time in the postpartum period. Approximately one‐third of women demonstrated adherence consistent with taking ≥2 doses/week in pregnancy and early postpartum and only one‐fifth did by months 6–12 postpartum, based on established thresholds [12]. In a subset of sexually active pregnant and postpartum women, and in breastfeeding women, recent adherence was higher than those with low or no sexual activity or non‐breastfeeding postpartum women. Women may adjust their daily PrEP use based on their actual risk/exposure and should be supported in this approach. We identified that older age, being pregnant versus postpartum, breastfeeding, having a partner living with HIV or of unknown serostatus and recent sex frequency (having sex 5+ times in the past month) were associated with taking any PrEP or levels consistent with taking PrEP ≥2 times per week. As women pass through various periods of risk during pregnancy and postpartum, it is appropriate to adjust their adherence and use of PrEP as in other populations, such as men who have sex with men [23, 24, 25]. Interventions that guide risk assessments to actual exposures and enable women to accurately make such assessments and choices are urgently needed.

We also found that self‐reports of PrEP use in the past 7 or 30 days did not accurately reflect objective levels of PrEP use. Objective measures of PrEP use will be essential for effective clinical practice. Our previous study demonstrated high levels of PrEP initiation in pregnancy (84% of women enrolled), and high continuation (66% returned for refill at 1 month and 58% at 3 months) [15]. However, our current sub‐study of objective adherence in the same cohort highlights the importance of utilizing objective measures, including TFV‐DP in DBS, plasma or hair, to monitor PrEP adherence, especially in PBFW.

Prior research has demonstrated that PrEP continuation during the peripartum period may be suboptimal [26]. Motivation to take PrEP during pregnancy may be high, especially among higher‐risk women with partners of unknown serostatus or living with HIV, and may decline in the postpartum period during periods of abstinence from sex, or when risk perception changes after weaning and no longer concerned with vertical HIV transmission [15, 27]. Our study demonstrated that women who reported recent sexual activity had double the odds of taking PrEP, which is important for understanding the drivers of preventive adherence [28, 29, 30].

Adherence to daily oral PrEP is complicated in pregnancy and postpartum, and PrEP continuation may wane during the postpartum period due to less frequent contact with the health facility. Due to known PrEP adherence and continuation barriers and pharmacokinetics of oral TDF‐FTC among PBFW [12], high‐risk PBFW who recognize their HIV risk could be ideal candidates for novel, long‐acting PrEP agents with less frequent dosing options. It is important that drug licenses and guidelines allow vaginal ring use or injectable PrEP in pregnancy for women who find daily use difficult [8]. In addition, given the high risk of HIV acquisition during pregnancy and postpartum combined with the suboptimal adherence measurements already described, there is a need for an objective, cumulative approach to measuring PrEP adherence both to improve adherence and reduce the risk of HIV acquisition. We recommend future studies on objective, cost‐effective ways to monitor PrEP adherence, including urine TFV monitoring (that measures recent PrEP use) [31, 32, 33] or periodic DBS surveys in peripartum to inform PrEP adherence interventions. Finally, there is a need for additional mixed‐methods research to explore further the barriers to taking PrEP daily and reasons for perceived reduced risk during postpartum, including why women may use PrEP intermittently.

Limitations of our study include potential overestimation of the true proportion of women who took PrEP as we excluded women who reported not taking PrEP in the past month. Further, we did not collect haematocrit, which is usually low and variable in pregnant and postpartum women and may underestimate TFV‐DP [20, 34].

## 4 CONCLUSIONS

This study sheds light on the PrEP use patterns and drivers of low PrEP use among women during pregnancy and into postpartum and identifies opportunities for interventions to promote daily PrEP use during periods of sexual activity. Clinical decisions should not depend upon self‐reported adherence. Focusing adherence interventions on pregnant and postpartum women at risk remains essential in ensuring optimal PrEP coverage prior to and during periods of exposure.

## COMPETING INTERESTS

DJD declares funding from ViiV as a consultant for a research meeting, and research funding from Gilead. The PrEP‐PP study received test kits from Cepheid for STI tests and Truvada (study drug) from Gilead.

## AUTHORS’ CONTRIBUTIONS

DJD conceptualized the study, conducted analysis and data collection, wrote the first draft and approved the final drafts of the manuscript.

DCN conducted data cleaning, analysis and writing of methods and results, and approved the final draft of the manuscript.

JCM reviewed the methodology of analysis, reviewed data analysis and methods, and approved the final draft of the manuscript.

LW conducted lab analysis, reviewed the methodology of analysis, reviewed data analysis and methods, and approved the final draft of the manuscript.

JN conducted lab analysis, reviewed the methodology of analysis, reviewed data analysis and methods, and approved the final draft of the manuscript.

RM conducted data cleaning, analysis and writing of methods and results, and approved the final draft of the manuscript.

NM coordinated the study, data collection and lab analysis, and approved the final draft of the manuscript.

LFJ reviewed the methodology of analysis, reviewed data analysis and methods, and approved the final draft of the manuscript.

LGB reviewed the methodology of analysis, reviewed data analysis and methods, and approved the final draft of the manuscript.

PG reviewed the methodology of analysis, reviewed data analysis and methods, and approved the final draft of the manuscript.

TJC conceptualized the study, reviewed and revised drafts of the analysis, and approved the final draft of the manuscript.

LM conceptualized the study, monitored the study implementation, reviewed and revised drafts of the analysis, and approved the final draft of the manuscript.

## FUNDING

DJD, TC, LFJ and LM received funding from the National Institute of Mental Health (R01MH116771) and Eunice Kennedy Shriver National Institute of Child Health and Human Development (R01HD106821). DLJD received funding from Fogarty International Center (K01TW011187). The University of Cape Town Clinical PK Laboratory is supported in part via the Adult Clinical Trial Group (ACTG), by the National Institute of Allergy and Infectious Diseases (NIAID) of the National Institutes of Health under award numbers UM1 AI068634, UM1 AI068636 and UM1 AI106701; as well as the Infant Maternal Pediatric Adolescent AIDS Clinical Trials Group (IMPAACT), funding provided by the National Institute of Allergy and Infectious Diseases (U01 AI068632), The Eunice Kennedy Shriver National Institute of Child Health and Human Development, and the National Institute of Mental Health grant AI068632.

## Data Availability

The data that support the findings of this study are available from the corresponding author upon reasonable requests.
